# Coverage optimization and node minimization in WSNs: an enhanced hybrid PSO approach with spatial position encoding

**DOI:** 10.1038/s41598-025-09849-4

**Published:** 2025-07-13

**Authors:** Yinghua Tong, Lianhai Lin, Liqin Tian, Zhigang Wang, Wenxing Wu, Junyi Wu

**Affiliations:** 1 School of Computer Science, Qinghai Normal University, Xining, 810016 Qinghai China; 2 School of Computer Science, North China Institute of Science and Technology, Langfang, 065201 Hebei China

**Keywords:** Wireless sensor networks (WSNs), Coverage optimization, Particle swarm optimization (PSO), Node deployment, Dynamic node adjustment, Environmental sciences, Computational science

## Abstract

Wireless sensor networks (WSNs) are widely used in various applications requiring efficient coverage and minimal resource utilization. This paper presents an enhanced hybrid particle swarm optimization (EHPSO) algorithm that incorporates a spatial position encoding (SPE) strategy to optimize coverage while dynamically adjusting the number of sensors deployed in WSNs. The proposed approach leverages the strengths of particle swarm optimization (PSO) by integrating it with the SPE mechanism, which effectively guides the search process towards high-quality solutions. The EHPSO algorithm is designed to balance exploration and exploitation capabilities, enabling dynamic node adjustment and ensuring robust performance across different network configurations and environmental conditions. Extensive simulations are conducted to evaluate the performance of the proposed method against state-of-the-art algorithms in terms of coverage quality and node count. A multi-objective optimization model is also established, further illustrating the algorithm’s performance and its effectiveness in balancing the number of sensors and coverage rate. Results demonstrate improvements in coverage optimization and reduction of node deployment compared to existing methods. This research contributes to more efficient and cost-effective deployment strategies for WSNs, particularly in scenarios where resources are limited and optimal coverage is critical.

## Introduction

The WSNs constitute a sophisticated system composed of numerous miniature sensor nodes that autonomously collaborate within designated areas to accomplish intricate monitoring and data collection tasks, enabling seamless information transmission and processing without the need for manual intervention^1^. Each sensor node is endowed with the capability not only to gather data from the environment or machinery but also to communicate with other nodes through wireless communication, forwarding this information to more distant receivers or directly to a central processing unit^2^. In recent years, propelled by advancements in automation technology, distributed information processing, and embedded systems, the application scope of WSNs has significantly broadened. These networks have found extensive application in critical fields such as continuous monitoring of complex and hazardous mining environments to ensure safety during extraction processes^3^; in emergency communication scenarios where traditional infrastructure has been compromised, such as in the aftermath of natural disasters, WSNs can rapidly establish temporary communication networks to guarantee timely information dissemination^4^; and in smart agriculture, where deploying WSNs facilitates precise management of irrigation systems and crop growth monitoring, thereby enhancing crop yields while reducing costs^5^.

The coverage area is a critical metric for evaluating the sensing capability of WSNs over their designated monitoring regions. An ideal coverage extent ensures that the network can effectively collect the required data, which is essential for maintaining the functionality and reliability of the WSN. Since sensor nodes are often deployed randomly, their positions may not always be optimal, necessitating the adjustment of node locations to enhance coverage density^6^. In practical applications, the effective sensing range between sensor nodes can be influenced by various factors, including but not limited to ambient white noise, humidity levels, and path loss during signal transmission^7^. Particularly under extreme conditions, such as underwater environments, signals may experience additional attenuation, surface noise, or significant propagation delays over long distances, all of which can alter the actual sensing range between nodes^8^. Additionally, the performance of the sensors themselves, including internal thermal noise, energy consumption, and remaining battery life, can impact the sensing accuracy. Therefore, rational planning of node deployment to accommodate these variations in sensing radius is a core strategy for improving the overall quality of coverage in WSNs. This not only aids in enhancing the effectiveness of data collection but also extends the operational lifespan of the network to some extent.

Research on the coverage of WSNs is a current hotspot due to its wide-ranging applications. In recent years, many scholars have conducted comprehensive reviews of WSN coverage studies^9–13^. Notably, Egwuche et al.^9^ focused on WSN, coverage optimization, and the application of machine learning and nature-inspired algorithms; Deng et al.^10^ provided an in-depth and comprehensive summary and categorization of coverage optimization problems and technologies based on data fusion; Priyadarshi et al.^11^ categorized various coverage techniques into four main sections: computational geometry-based techniques, force-based techniques, grid-based techniques, and meta-heuristic techniques, and reviewed them accordingly; Ahmad et al.^12^ reviewed the issue of sensor positioning in the context of WSN coverage; Abdulwahid et al.^13^ provided an overview from the perspective of smart cities.

According to Priyadarshi et al.^11^, research methodologies concerning WSN coverage optimization can be categorized into traditional algorithms and meta-heuristic approaches; traditional methods encompass computational geometry-based techniques, force-based techniques, and grid-based techniques. Specifically, for computational geometry-based approaches, Yu et al.^14^ addressed the k-coverage problem by proposing centralized and distributed protocols that utilize the concept of coverage contribution area to minimize sensor spatial density while considering residual energies. Another study by Liao et al.^15^ tackled the problem by decomposing it into two sub-problems, proposing heuristic algorithms for the target coverage problem and an efficient solution for network connectivity based on the Steiner minimum tree and Voronoi partition. For force-based techniques, Luo et al.^16^ introduced a 3D virtual force coverage algorithm to redeploy drifted underwater nodes for improved coverage, with simulations indicating enhanced coverage rates compared to existing algorithms. Mahboubi et al.^17^ developed efficient deployment algorithms using virtual forces derived from Voronoi diagrams to increase the coverage area iteratively. For grid-based techniques, Liu et al.^18^ proposed a virtual square grid-based coverage algorithm that divides the sensing range into virtual grids, achieving better performance with fewer active nodes and lower computational complexity.

The use of meta-heuristic methods to solve this optimization problem has become a recent hot topic, different optimization objectives lead to distinct categories of research. Focusing solely on maximizing coverage, most studies consider the complexity associated with traditional algorithms too high and recognize that meta-heuristic algorithms present a challenging problem to solve. Jin et al.^19^ improved the marine predator algorithm, introducing an enhanced marine predator algorithm. Ou et al.^20^, taking symmetry into account when deploying nodes to simplify problem-solving, proposed an improved Grey Wolf Optimizer (GWO) with multi-strategies. Wang et al.^21^, addressing the coverage problem in a 3D plane, presented an enhanced GWO.

In recent years, the Mobile Sensor Deployment (MSD) problem has become a novel area of study in the optimization of coverage for WSNs, employing meta-heuristic techniques. Liao et al.^15^ were the first to define this problem, which essentially involved optimizing the movement distance of initially deployed mobile sensor nodes to minimize deployment costs while maximizing coverage. In the study of MSD problem, Wen et al.^22^ divided the problem-solving process into two phases, utilizing the vampire bat algorithm to solve cellular grid division and then applying an improved virtual force algorithm to derive the final solution. Li et al.^23^ approached the problem using multi-objective optimization, improving the classic multiobjective ant lion optimization (MOALO) algorithm and proposing an enhanced MOALO algorithm based on fast nondominated sorting. Additionally, Li et al.^24^ also considered employing an improved sand cat swarm optimization algorithm to solve the problem. Wu et al.^25^ introduced a multi-objective optimization algorithm for WSNs based on an improved PSO, in each iteration, they calculated the population fitness values and compared these with historical best values to prevent the loss of the optimal solution.

In addition to the MSD problem mentioned earlier, coverage issues in WSNs are often discussed alongside other optimization problems, forming joint optimization models. Dong et al.^26^, inspired by the foraging behavior of krill, improved the movement pattern of krill swarms and applied it to sensors with distributed communication, simultaneously optimizing energy consumption and coverage. Dananjayan et al.^27^, drawing on the artificial bee colony algorithm, proposed a method to reduce the number of sensors used in agricultural settings and extend the lifespan of sensor nodes by leveraging bio-inspired energy-saving protocols. Nematzadeh et al.^28^ introduced a meta-heuristic algorithm based on the GWO to ensure connectivity by generating topology graphs, thereby maximizing coverage while maintaining the connectivity of WSNs. Kumar De et al.^29^ considered the joint optimization of three objectives in WSNs: maximizing coverage area, minimizing coverage holes, and reducing energy demand, proposing an algorithm that combines genetic algorithms and PSO.

In summary, traditional methods suffer from high computational complexity, leading to rapid node depletion due to high energy consumption, which can result in coverage holes and fail to achieve the desired network coverage in WSNs. Meta-heuristic optimization methods, based on biomimetic algorithms, can significantly enhance network connectivity and coverage. However, existing meta-heuristic algorithms tend to converge prematurely, frequently getting trapped in local optima with high computational complexity, affecting overall coverage. This trend can also lead to partial coverage gaps and redundant nodes, diminishing network coverage efficiency.

Currently, there are many studies based on meta-heuristics, but these studies have the following issues:

1. Due to the characteristics of meta-heuristic algorithms, when using traditional encoding, if the number of sensors is relatively large (for example, more than 50), the algorithm may struggle to find better solutions due to chaotic search problems.
2. Existing research primarily focuses on optimizing sensor coverage to reduce the number of sensors. However, during the actual optimization process, the optimal number of sensors is not explicitly discussed; instead, the coverage of the sensor network is discussed with a predetermined number of sensors. Yet, the quantity of sensors has a decisive impact on the operational costs of the sensor network.
3. In many ecological monitoring scenarios, there are areas where sensors cannot be deployed or regions within the entire monitoring area that do not require monitoring. These areas need to be considered and avoided in the optimization process.

In response to these issues, this paper discusses the target coverage problem in WSNs, establishing a model that minimizes the number of sensor nodes while ensuring coverage, thereby reducing deployment costs. Our proposed strategy’s contributions compared to existing methods are outlined in Table 1.

**Table 1:** Comparison of related works.

Ref.	Optimization objective	Method
^19^	COVR	Enhanced marine predator
^20^	COVR	Improved GWO
^21^	COVR (3D)	Enhanced GWO
^22^	COVR and moving distance	Vampire bat and improved virtual force
^23^	COVR and moving distance	MOALO
^24^	COVR and moving distance	Improved sand cat
^25^	COVR and moving distance	Improved PSO
^26^	COVR and energy consumption	Krill herd
^27^	Lifespan and number of sensors	Artificial bee colony algorithm
^28^	COVR and connectivity	Enhanced GWO
^29^	COVR and energy consumption	PSO and genetic algorithms
This work	COVR and number of sensors	Enhanced hybrid PSO

In this paper, we propose an algorithm named EHPSO, which is based on the PSO algorithm. The main contributions of this paper are as follows:

- A coding strategy SPE is proposed, which can further enhance the coverage of WSNs.
- Considering the target coverage problem in WSNs, the EHPSO algorithm is proposed, capable of adjusting the number of sensor nodes during optimization.
- Comparative analysis is performed against state-of-the-art algorithms to highlight the advantages of the proposed algorithm.
- A multi-objective optimization model is established to further evaluate the performance of the EHPSO algorithm in minimizing the number of nodes and maximizing coverage.
- Experiments are conducted under different scenarios to analyze the optimization performance of the algorithm, and the impact of Non-Critical Areas (NCA) and Restricted Areas (RA) on the deployment of the sensor network is also investigated.

The rest of the paper is organized as follows: “Problem formulation” describes the problem formulation. “EHPSO algorithm” details the standard PSO and the EHPSO. “Simulation experiments” presents the experimental setup and results. “Conclusion and future work” concludes the article and discusses future work.

## Problem formulation

In this section, we present the coverage models and optimization equations considered in our study.

### Coverage model

**Fig. 1:**
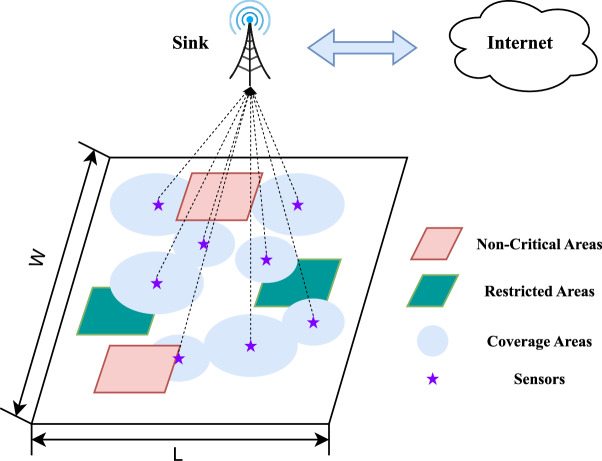
Coverage model of sensor network including NCA and RA regions.

We consider a monitoring area of size $L \times W$, which is divided into $L \times W$ grids, as shown in Fig. 1. We assume that *N* sensors are to be deployed within this area. The sensor data collected by these sensors is transmitted to the Internet via a Sink node, thereby enabling information transfer. In this process, we focus on both the coverage rate of the entire area and the number of deployed sensors.

For coverage, we use the disk coverage model^10^. The Euclidean distance between sensor $S_i$ and grid $G_j$ is denoted as $\Vert S_i,G_j\Vert$. Whether $G_j$ is covered by $S_i$ can be expressed as follows:

1 $$\begin{aligned} \text {C}(S_i,G_j) = {\left\{ \begin{array}{ll} 1, \Vert S_i,G_j\Vert \le R_i \\ 0, \Vert S_i,G_j\Vert > R_i \end{array}\right. }, \end{aligned}$$

where $R_i$ represents the sensing radius of $S_i$. In this paper, the communication radius of $S_i$ is set as $2R_i$, to ensure the connectivity of the WSN^19^. Further, whether grid $G_j$ is covered can be represented as follows:

2 $$\begin{aligned} \text {C}(G_j) = {\left\{ \begin{array}{ll} 1, \forall i, \Vert S_i,G_j\Vert \le R_i \\ 0, \forall i, \Vert S_i,G_j\Vert > R_i \end{array}\right. }. \end{aligned}$$

Then the coverage rate for the entire monitoring area can be expressed as follows:

3 $$\begin{aligned} \text {COVR}(S,G) = \frac{\sum _{\forall j}\text {C}(G_j)}{L \times W}, \end{aligned}$$

where *S* denotes the sensors set, *G* denotes the grids set.

### Optimization objective

When only considering the coverage rate, the problem can be formulated as:

4 $$\begin{aligned} \max _{\{X, Y\}} \text {COVR}(S,G), \end{aligned}$$

where $\{X, Y\}$ denotes the coordinates of the sensors. This optimization objective is referred to as OBJ-I.

Considering the practical constraints in monitoring tasks, there exist areas where sensors cannot be deployed and regions that do not require consideration. Therefore, we introduce the concepts of NCA and RA, as depicted in Fig. 1:

1. NCA refers to areas that do not require sensor coverage, such as safe zones during emergency rescue operations or uninhabited areas.
2. RA refers to areas that require sensor coverage but where sensors cannot be deployed, such as nature reserves or historical buildings.

Furthermore, aiming to reduce the number of deployed sensors and thus minimize deployment costs, we propose an additional optimization equation, OBJ-II:

5 $$\begin{aligned} \begin{aligned}&\min _{\{X, Y\}} N + \epsilon \times (-\text {COVR}(S,G)) \\ \text {s.t. }&\text {COVR}(S,G) \ge C_{obj} \\&\forall j, G_j \notin \text {NCA} \\&\forall i, S_i \notin \text {RA} \end{aligned}, \end{aligned}$$

where $C_{obj}$ represents the requirement coverage rate, $\epsilon$ is a very small positive value used to ensure that the minimization of *N* takes priority over the maximization of $\text {COVR}(S,G)$.

Finally, when constructing the multi-objective optimization model, the optimization equation is OBJ-III:

6 $$\begin{aligned} \begin{aligned}&\min _{\{X, Y\}} \{N, -\text {COVR}(S,G)\} \\ \text {s.t. }&\text {COVR}(S,G) \ge C_{obj} \\&\forall j, G_j \notin \text {NCA} \\&\forall i, S_i \notin \text {RA} \end{aligned}. \end{aligned}$$

## EHPSO algorithm

This section introduces the proposed EHPSO algorithm. First, we will present the motivation behind EHPSO, followed by a detailed description of its implementation.

### Motivation

As discussed in the Introduction section, meta-heuristic-based algorithms face several challenges in the current problem: they tend to suffer from search chaos when applied to large-scale problems; sensor number optimization has rarely been studied; and the discussion on NCA and RA areas is insufficient. In particular, regarding the second issue, there are almost no traditional optimization methods that can effectively address it. We are especially interested in how to reduce deployment costs while ensuring sensing coverage. To achieve this, we considered modifying the PSO algorithm into a single-solution-based approach^30,31^, which allows for more flexible adjustment of the individual’s encoding length, thereby enabling the optimization of sensor numbers.

**Fig. 2:**
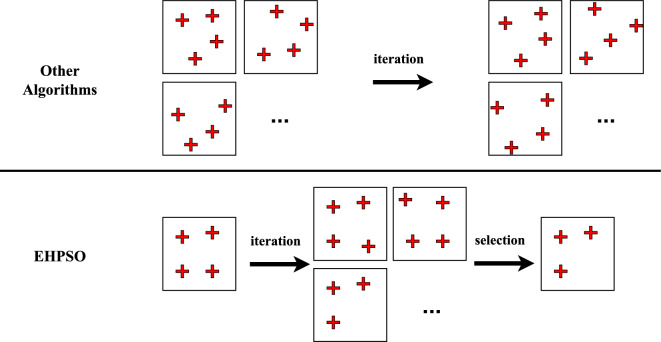
The evolutionary mechanism in the EHPSO.

Figure 2 illustrates the conceptual idea of the evolutionary mechanism in the EHPSO algorithm. In traditional PSO, each particle evolves based on its own and the global best solution. However, this strategy does not perform well in high-dimensional problems. Instead, we adopt a single-solution-based approach, where only one effective solution is maintained during the entire optimization process. In each iteration, multiple offspring are generated based on this single solution. We believe this evolutionary strategy is better suited for solving high-dimensional problems, which motivated the design of the EHPSO algorithm.

### Initialization method

A good initialization algorithm can significantly simplify the coverage optimization problem. Inspired by^22^, this paper proposes an initialization method based on Cellular Grid Division (CGD). Additionally, considering the optimization objective OBJ-II proposed in this paper, we develop the EHPSO algorithm, which is based on the PSO algorithm; and considering the multi-objective OBJ-III proposed in this paper, we develop the multi-objective particle swarm optimization (MOPSO) algorithm.

The CGD method is a highly effective approach for initializing sensor deployment. However, this method determines the number of sensors based on the task area and sensing radius, making it difficult to specify the number of sensors for deployment, which limits its applicability. To address this limitation, this paper assumes a square task area (i.e., $L = W$) and proposes a more flexible initialization deployment scheme, as follows:

Step 1: Calculate the number of sensor nodes in each row, $A_1=\lceil \sqrt{N} \rceil$. The total number of rows is $A_2=\lfloor N/\lceil \sqrt{N} \rceil \rfloor$, and the distance between nodes in each row is $A_3=L/A_1$.

Step 2: For each sensor $S_i$ in all rows except the last row, set the X and Y coordinates using the following formulas:

7 $$\begin{aligned} {\left\{ \begin{array}{ll} x_i = (0.25 + 0.5 \times (i~\text {mod}~2)) \times A_3 \\ y_i = (i-0.5) \times C \times (A_2/A_1) \end{array}\right. }, \end{aligned}$$

where $A_2/A_1$ is used to proportionally scale the Y-coordinates.

Step 3: To allow for more flexible node deployment, we make special arrangements for the last row to ensure that the nodes in the last row are also evenly distributed across the space. Set the X and Y coordinates of the nodes in the last row using the following formulas:

8 $$\begin{aligned} {\left\{ \begin{array}{ll} x_i = (i - (N - A_1 \times A_2) + 0.5) \times \frac{L}{N - A_1 \times A_2} \\ y_i = (A_1 - 0.5) \times A_3 \end{array}\right. }. \end{aligned}$$

This initialization method yields results similar to those of the CGD method but allows for different deployment configurations based on the number of sensors.

### Adaptive evaluation strategy

**Fig. 3:**
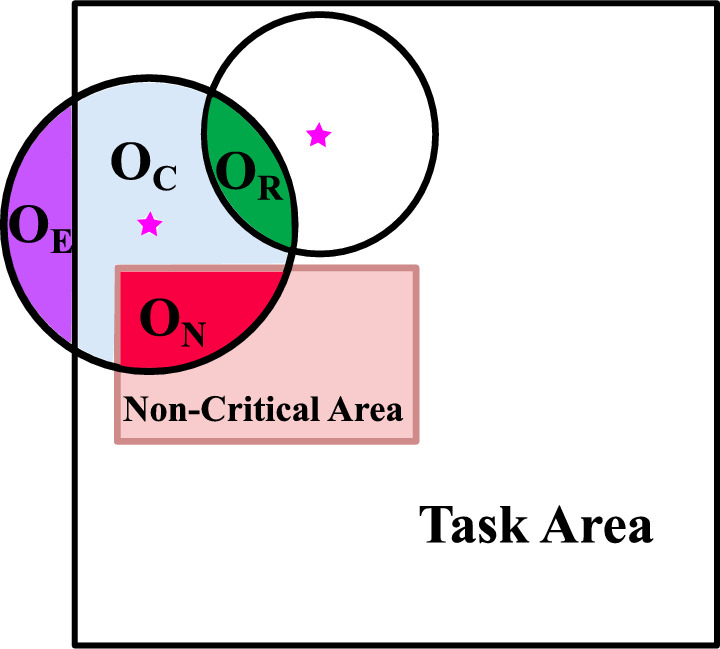
Division of different areas.

Considering that sensor ranges may overlap or extend beyond the task area, we propose an adaptive evaluation strategy. As shown in Fig. 3. We divide the sensing range into four regions:

- $\text {O}_\text {C}$: the area of the covered region.
- $\text {O}_\text {E}$: the area of the region exceeding the task area.
- $\text {O}_\text {N}$: the area overlapping with NCA.
- $\text {O}_\text {R}$: the area overlapping with the sensing ranges of other sensors.

In our fitness evaluation function, the coverage area of sensor $S_i$ is expressed as:

9 $$\begin{aligned} \text {COVR}(S_i) = \text {O}_\text {C} - \alpha (\text {O}_\text {E} + \text {O}_\text {N} + \text {O}_\text {R}), \end{aligned}$$

where $\alpha$ is a parameter that linearly decreases with the number of iterations. Its initial value is 0.5, and it becomes 0 when the iteration *t* reaches half of the maximum number of iterations $T_{\text {max}}$:

10 $$\begin{aligned} \alpha = {\left\{ \begin{array}{ll} 0.5 - \frac{t}{T_{max}} & \text {if } t \le 0.5 \times T_{\text {max}} \\ 0 & \text {if } t > 0.5 \times T_{\text {max}} \end{array}\right. }. \end{aligned}$$

This adaptive evaluation strategy helps to balance the coverage area while penalizing excessive overlap and out-of-bound regions, thereby optimizing the overall network coverage.

### Standard PSO

The PSO algorithm draws inspiration from the social behavior of bird flocks^32^. In PSO, each individual in the population, referred to as a particle, shares information about the global best solution and continuously updates its position to converge towards an optimal solution. This process involves each particle refining its solution iteratively, leading the entire swarm to evolve from a disordered state to a more organized one, ultimately converging on the most optimal solution to the problem. Currently, the PSO algorithm is widely applied in various fields, including networking, robotics, image segmentation, power generation and controlling, fuzzy systems and so on^33^.

For the WSN coverage problem, meta-heuristic algorithms like PSO typically use a population of particles represented as follows:

11 $$\begin{aligned} P = \begin{bmatrix} x_{1,1} & y_{1,1} & x_{1,2} & y_{1,2} & \cdots & x_{1,N} & y_{1,N} \\ x_{2,1} & y_{2,1} & x_{2,2} & y_{2,2} & \cdots & x_{2,N} & y_{2,N} \\ \vdots & \vdots & \vdots & \vdots & \ddots & \vdots & \vdots \\ x_{M,1} & y_{M,1} & x_{M,2} & y_{M,2} & \cdots & x_{M,N} & y_{M,N} \end{bmatrix}, \end{aligned}$$

where *M* is the number of individuals in the population.

In each iteration, particles adjust their velocities based on their own best historical positions and the global best position. The velocity update formula is:

12 $$\begin{aligned} \begin{aligned} v_{i,j}(t+1)&= w \times v_{i,j}(t) \\&\quad + c_1 \times r_1 \times (pbest_{i,j} - x_{i,j}(t)) \\&\quad + c_2 \times r_2 \times (gbest_j - x_{i,j}(t)) \end{aligned}, \end{aligned}$$

where $pbest_{i,j}$ is the historical best position of the *j*-th sensor in the *i*-th individual, and $gbest_j$ is the global best position of the *j*-th sensor.

The position update formula is:

13 $$\begin{aligned} x_{i,j}(t+1) = x_{i,j}(t) + v_{i,j}(t+1). \end{aligned}$$

Although the PSO algorithm, due to its simplicity and ease of parallelization, has been widely applied in many fields, it has certain limitations when applied to WSN coverage problems. Specifically, the WSN coverage problem is inherently a point-finding problem, where the X and Y coordinates are interrelated. However, the standard PSO algorithm does not capture this relationship. To address this issue, we propose a SPE coding strategy and improve the PSO algorithm accordingly.

### SPE and EHPSO algorithm

The WSN coverage problem discussed in this paper is a two-dimensional problem involving only the X and Y coordinate axes. Each sensor node possesses two-dimensional spatial attributes and is relatively independent of others. Based on these considerations, we designed the SPE method. In the SPE encoding, each particle represents the position and velocity of a sensor node, and the entire population represents an effective deployment scheme. Specifically, the population *P* is defined as:

14 $$\begin{aligned} P = \begin{bmatrix} x_1 & y_1 & v_{x_1} & v_{y_1} \\ x_2 & y_2 & v_{x_2} & v_{y_2} \\ \vdots & \vdots & \vdots & \vdots \\ x_N & y_N & v_{x_N} & v_{y_N} \end{bmatrix}, \end{aligned}$$

where $x_i$, $y_i$, $v_{x_i}$, and $v_{y_i}$ represent the X-coordinate, Y-coordinate, X-axis velocity, and Y-axis velocity of sensor node $S_i$, respectively.

The update formula for $v_{x_i}$ (with a similar formula for $v_{y_i}$) is:

15 $$\begin{aligned} v_{x_i}(t+1) = {\left\{ \begin{array}{ll} v_{x_i}(t) & \text {fit}(x_i(t+1)) > \text {fit}(x_i(t)) \\ \text {rand}(-V, V) & \text {otherwise} \end{array}\right. }, \end{aligned}$$

where fit is the fitness evaluation function, *V* represent the max velocity in current iteration and $\text {rand}(-V, V)$ is a random real number from $-V$ to *V*.

The position update formula is:

16 $$\begin{aligned} {\left\{ \begin{array}{ll} x_i(t+1) = x_i(t) + v_{x_i}(t+1) \\ y_i(t+1) = y_i(t) + v_{y_i}(t+1) \end{array}\right. }. \end{aligned}$$

Since the entire population in PSO represents a deployment scheme for the sensors, the optimal number of sensors can be found by adjusting the population size. The flow of the EHPSO algorithm is outlined in Algorithm 1.

**Algorithm 1:**
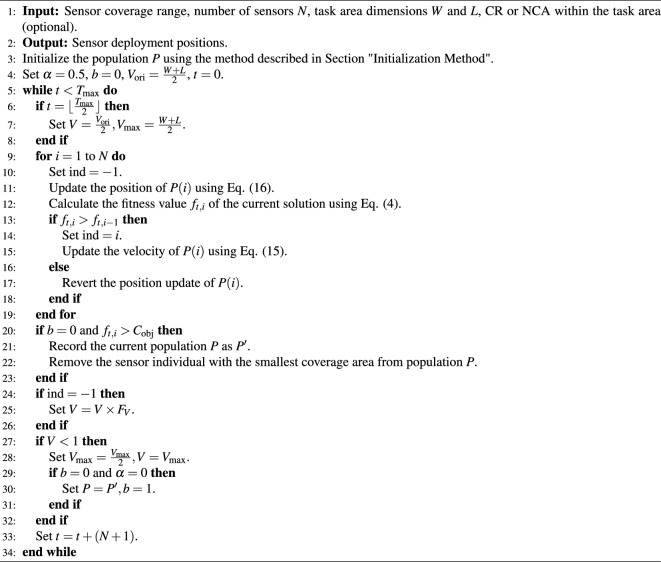
EHPSO algorithm.

In Algorithm 1, $b$ is a flag indicating whether the optimization of the number of sensors has been completed. $b = 0$ indicates that the optimization of the number of sensors has not been completed. $F_V$ is the decay parameter for the velocity, used in conjunction with the parameter $V$ to control the overall movement speed of the particles. In this paper, the value of $F_V$ is set to 0.9. The entire algorithm can be divided into seven steps:

Step 1: Initialization: Use the method described in Section “Initialization method” to obtain an initial deployment scheme.

Step 2: Midpoint Check: Determine whether the algorithm has reached the midpoint of the maximum number of iterations ($T_{\text {max}}$). If it has, set the parameter $\alpha$ to 0 and reduce the maximum velocity $V$ to half of its original value to perform a more detailed search.

Step 3: Population Traversal: Iterate through the entire population. For each individual, update its position and calculate its fitness. Retain the individual with a higher fitness.

Step 4: Remove a Sensor: If the optimization of the number of sensors has not been completed ($b = 0$) and the population’s fitness meets the minimum coverage requirement, record the current population as $P'$ and remove the sensor node with the smallest coverage area.

Step 5: Velocity Update: If no better population is found in the current iteration, update the maximum velocity $V$ to $V \times F_V$.

Step 6: Local Optimum Handling: If $V$ is less than 1, it indicates that the algorithm has fallen into a local optimum. Reset $V$ to half of its original maximum value and perform a new search. If $\alpha = 0$ and $b = 0$, it means that the optimization of the number of sensors has not been completed and the current coverage does not meet the minimum requirement. Therefore, revert the population $P$ to $P'$, set $b = 1$ to indicate that the optimization of the number of sensors has been completed, and proceed with further coverage optimization using the optimal number of sensor nodes.

Step 7: Iteration: Repeat steps 2 to 6 until the maximum number of iterations ($T_{\text {max}}$) is reached.

This approach ensures that the WSN coverage problem is effectively addressed by dynamically adjusting the number of sensors and optimizing their deployment positions to achieve the desired coverage.

### Multi-objective optimization

The two optimization objectives of number of sensors and coverage rate cannot be optimized simultaneously, forming a multi-objective optimization problem^34^. Considering that the proposed SPE encoding makes each population in the algorithm correspond to a deployment plan, it is necessary to construct multiple populations to achieve multi-objective optimization. Based on our proposed EHPSO algorithm, we have designed MOPSO, whose optimization target is OBJ-III. The steps of the algorithm can be described as follows:

Step 1: Initialize the deployment of *K* populations simultaneously using the initialization algorithm described in Section “Initialization method”.

Step 2: For each population, For each population, check whether the current iteration has reached the midpoint of the maximum number of iterations $T_{max}$. If so, set the parameter $\alpha$ to 0 and reduce the maximum velocity $V$ to half its original value, allowing for a more refined search in later stages. Then, perform a full traversal of the population: update each individual’s position and evaluate its fitness, retaining individuals with higher fitness values.

Step 3: Perform non-dominated sorting on the deployment plans corresponding to all populations, and calculate the crowding distance based on the size of the population (i.e., the number of sensors) and coverage rate. Then, in conjunction with step 4 of Algorithm 1, recalculate the crowding distance after performing deletion operations on each population, retaining the populations with larger crowding distances.

Step 4: For each population, update the maximum velocity $V$ if no better solution is found during the current iteration. Specifically, multiply $V$ by a factor $FV$. If $V$ drops below 1, indicating that the algorithm may be trapped in a local optimum, reset $V$ to half of its initial maximum value and initiate a new search. Additionally, if $\alpha = 0$ and $b=0$, revert the population to its previous state $P'$, set $b = 1$ to mark the completion of sensor number optimization, and continue optimizing coverage with the optimal number of sensors.

Step 5: Repeat steps 2 to 4 until the maximum iteration count $T_{\text {max}}$ is reached.

Due to the special nature of SPE encoding, to facilitate population iteration, we directly set the upper limit of the population size for multi-objective optimization to *K*, ensuring that the number of populations remains at *K* throughout subsequent iterations.

### Complexity analysis

During the execution of the EHPSO algorithm, the most computationally intensive part is the fitness evaluation, specifically the assessment of coverage for sensor deployment. According to our runtime statistics, this component accounts for over 99% of the total execution time. The complexity of the fitness evaluation can be expressed as $O(W \times L \times N)$, leading to an overall complexity of the EHPSO algorithm being $O(W \times L \times N \times T_{\text {max}})$.

Compared to other meta-heuristic based algorithms, such as EMPA^19^ and PSO^35^, where each individual represents a deployment scheme, it is necessary to evaluate the fitness of all individuals during one iteration. Therefore, the overall algorithm complexity is $O(N_p \times T_{iter} \times W \times L \times N)$, where $N_p$ denotes the population size, $T_{iter}$ denotes the number of iterations. For instance, in the study of the EMPA^19^, $N_p$ is 30 and $T_{iter}$ is 1000; in the study of the PSO mentioned above^35^, $N_p$ is 30 and $T_{iter}$ is 800. In this paper, the number of runs for the EHPSO algorithm is set to 1000*N*. Thus, when $N=30$, the complexity is comparable to similar algorithms; when $N>30$, it is slightly higher; and when $N<30$, it is slightly lower.

## Simulation experiments

Given the limited research focusing on optimizing OBJ-I, OBJ-II and OBJ-III, we designed four experiments to evaluate the performance of the proposed EHPSO algorithm.

Experiment 1: This experiment uses OBJ-I as the optimization objective and compares the EHPSO algorithm with other recently published algorithms. The goal is to assess the performance of EHPSO in typical WSN coverage problems. In this experiment, the EHPSO algorithm does not employ the dynamic node adjustment strategy.

Experiment 2: This experiment mainly conducts an ablation analysis of the proposed algorithm, analyzing the effectiveness of the proposed strategy in terms of optimization results and comparing it with the standard PSO algorithm.

Experiment 3: This experiment uses OBJ-II and OBJ-III as the optimization objective to evaluate the effectiveness of the EHPSO and the MOPSO algorithms in different scenarios. The aim is to demonstrate the EHPSO algorithm’s capability to optimize coverage under varying conditions.

Experiment 4: This experiment focuses on the impact of RA and NCA on the coverage performance of the EHPSO algorithm. The goal is to investigate how these factors influence the algorithm’s ability to achieve optimal coverage in complex environments.

The $T_{max}$ is set to 1000*N* for all three experiments and the value of *K* is set to 30 for multi-objective optimization. Both experiments were conducted on a Windows 10 operating system using the Matlab 2021b simulation environment.

### Experiment 1: comparison of EHPSO with other algorithms

To validate the performance of the EHPSO algorithm in WSN coverage problems, Experiment 1 compares the EHPSO algorithm with four recent meta-heuristic algorithms: EMPA^19^, IVFA^22^, PSO-MBO^36^ and COOTCLCO^37^. Among these four algorithms, the EMPA algorithm is the best-performing one. Since the EMPA algorithm does not incorporate an initialization strategy, which might introduce some unfairness to EHPSO, we designed a modified version, EMPAIS, which includes the initialization strategy proposed in this paper.

Three different scenarios were tested using these six algorithms: 1. The first two scenarios involve square task areas (i.e., $L = W$)^19^. 2. The third scenario involves a rectangular task area (i.e., $L \ne W$)^22^, with the sensor sensing radii following a normal distribution with a mean of 35 and a standard deviation of 6 (i.e., $R=\mathcal {N}(35, 6)$).

**Fig. 4:**
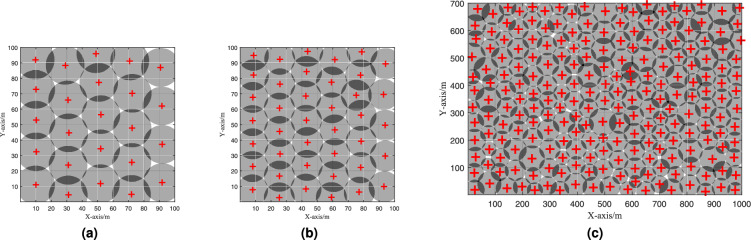
Coverage optimization effect of EHPSO in three scenarios. (**a**) $R=12.5, N=24$. (**b**) $R=10, N=40$. (**c**) $R=\mathcal {N}(35, 6), N=219$.

The experimental parameters and results are summarized in Table 2 and illustrated in Fig. 4.

**Table 2:** Experimental parameters and results.

Parameter	Scenario 1	Scenario 2	Scenario 3
Number of Sensors	24	40	219
W (m)	100	100	1000
L (m)	100	100	700
Sensor range (m)	12.5	10	$\mathcal {N}(35, 6)$

Algorithm	COVR (%)	COVR (%)	COVR (%)
EHPSO	**97.55**	**98.54**	**99.24**
EMPA	97.03	97.49	91.13
EMPAIS	97.29	98.10	96.18
IVFA	95.35	97.27	97.21
PSO-MBO	96.13	97.46	90.77
COOTCLCO	96.22	97.19	90.48
Initialization method	95.25	97.25	96.18

Significant values are in bold.

From the experimental results, it is evident that the EHPSO algorithm achieved the highest WSN coverage in all three scenarios. Notably, in the third scenario, the coverage rate reached 99.24%, which is an exceptionally high coverage rate. In the first and second scenarios, the IVFA algorithm did not show significant improvement over the initial solution. We attribute this to the characteristics of force-based algorithms, which tend to perform better in scenarios with fewer sensor voids.

In the third scenario, the EMPAIS algorithm achieved a coverage rate of 96.18%, which is equal to the coverage rate obtained by the initialization method. This is likely due to the high dimensionality of the problem caused by the large number of sensors, making it difficult to find an optimization direction using traditional encoding methods. In contrast, the EHPSO algorithm can flexibly adjust the positions of sensor nodes based on the initial configuration, thereby always finding a better evolutionary direction. Compared to the coverage rates obtained by EMPA and EMPAIS in the first two scenarios, the EMPAIS algorithm, which uses initialization, consistently outperformed the EMPA algorithm. This indicates that using an initialization strategy in conjunction with meta-heuristic algorithms can effectively enhance the optimization performance.

This experiment demonstrates the superior performance of the EHPSO algorithm in achieving high WSN coverage rates, particularly in complex scenarios with varying task areas and sensor distributions.

### Experiment 2: ablation study of proposed strategy

To evaluate the roles of the initialization method, the Adaptive Evaluation Strategy, and SPE encoding in the optimization process, we conducted an ablation study using traditional PSO and the proposed EHPSO. Considering that PSO has undergone multiple version iterations, we tested two versions of PSO: the PSO algorithm proposed in 1995 (denoted as PSO_1995)^38^ and the PSO algorithm proposed in 1998 (denoted as PSO_1998)^39^. The PSO_1998 algorithm uses a dynamic weight strategy (linearly decreasing weight strategy, with a maximum weight of 0.9 and a minimum weight of 0.2). Additionally, we improved PSO_1998 with the initialization strategy proposed in this paper, designing a new algorithm called PSO_1998_init. For EHPSO, we denote EHPSO without the initialization strategy as EHPSO_nInit and EHPSO without the adaptive evaluation strategy as EHPSO_nAdapt. The optimization results of these algorithms in three scenarios are shown in Table 3.

**Table 3:** Ablation study results.

Parameter	Scenario 1	Scenario 2	Scenario 3
EHPSO	**97.55**	**98.54**	**99.24**
EHPSO_nInit	97.54	98.52	96.36
EHPSO_nAdapt	97.54	98.54	97.48
PSO_1995	81.00	80.14	74.24
PSO_1998	95.04	97.27	90.41
PSO_1998_init	96.52	98.50	97.13

Significant values are in bold.

**Fig. 5:**
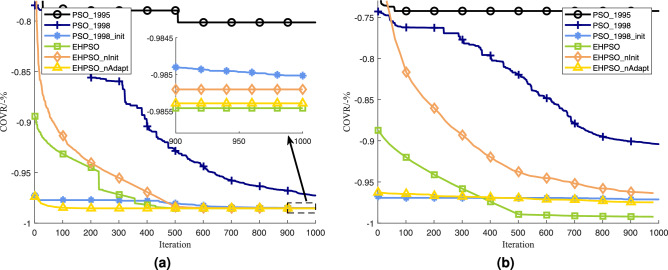
The iteration curves in Scenarios 2 and 3.

The iteration curves of the algorithms in Scenarios 2 and 3 are shown in Fig. 5. Note that since EHPSO and EHPSO_nInit use the adaptive evaluation strategy, their values during the first half of the iterations represent weighted coverage rates.

From Fig. 5 and Table 3, it can be observed that EHPSO achieves the best optimization performance. Standard PSO without dynamic weighting struggles to find the correct direction for evolution, while PSO with dynamic weighting converges relatively slowly. The comparison between PSO_1998 and PSO_1998_init demonstrates that for standard PSO, the convergence speed is relatively slow when solving large-scale WSN coverage optimization problems, but a good initialization method can effectively improve its convergence speed.

The comparison between EHPSO and EHPSO_nInit confirms the effectiveness of the initialization algorithm. The comparison between EHPSO and EHPSO_nAdapt confirms the effectiveness of the adaptive evaluation strategy proposed in this paper. The comparison between EHPSO_nInit and EHPSO_nAdapt indicates that when the number of sensors is small, an adaptive evaluation strategy is more important than a good initialization strategy. However, as the number of sensors increases, the importance of the initialization strategy grows significantly.

This ablation study clearly demonstrates the effectiveness of the proposed strategies in enhancing the performance of EHPSO.

### Experiment 3: evaluation of EHPSO under OBJ-II

**Fig. 6:**
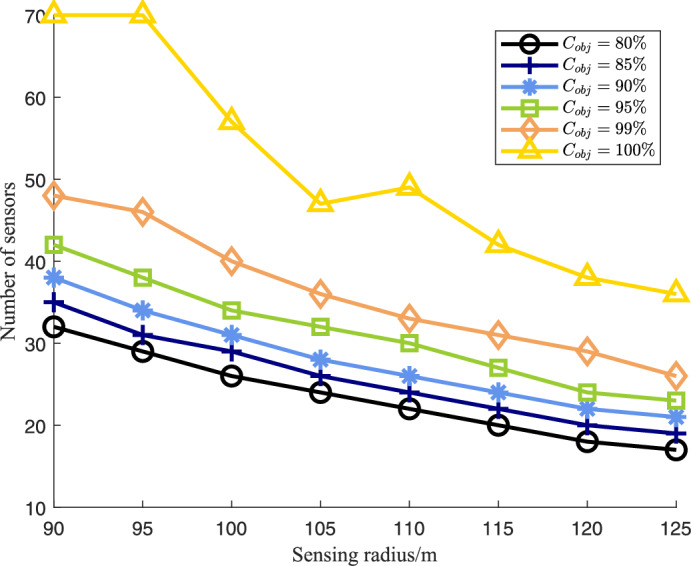
Sensing radius vs. number of sensors for different coverage requirements.

To evaluate the capability of the EHPSO algorithm to adjust the number of sensor nodes in different scenarios, we conducted five sets of experiments. Each set was configured with different coverage requirements, specifically 80%, 85%, 90%, 95%, and 99%. Within each set, experiments were performed using different sensing radii to investigate the relationship between sensing radius and the number of sensor nodes, as shown in Fig. 6.

According to the results presented in Fig. 6, increasing the sensing radius or the number of sensor nodes are both effective methods to improve the COVR of WSNs. The distances between the five curves in the figure vary, with a relatively larger gap between the curves for $C_{\text {obj}} = 100\%$ and $C_{\text {obj}} = 99\%$. This indicates that as the sensing radius increases, the required number of sensor nodes to achieve the desired coverage decreases. To further validate this conclusion, we analyzed the relationship between different coverage rates and the number of sensor nodes. The average additional number of sensors required to increase the coverage from 80% to 85% is 2.1, from 85% to 90% is 2.6, from 90% to 95% is 3.4, from 95% to 99% is 5.0, and from 99% to 100% is 15.0. These results suggest that as the target coverage rate increases, the number of additional sensors required to achieve each 1% increase in coverage also increases. Therefore, achieving 100% coverage with minimal cost remains a significant challenge and an area worthy of further research.

**Fig. 7:**
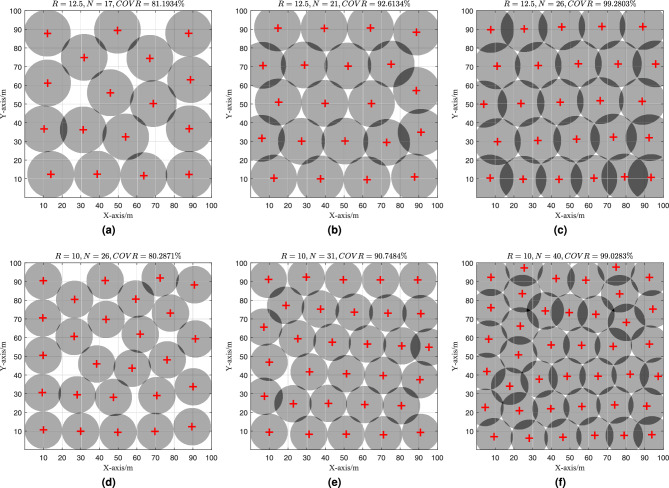
Coverage optimization effect of EHPSO. (**a**–**c**) $R=125, C_{obj}=80\%, 90\%$ and $99\%$, respectively. (**d**–**f**) $R=100, C_{obj}=80\%, 90\%$ and $99\%$, respectively.

To further analyze the performance of the EHPSO algorithm, we visualized some of the experimental results, as shown in Fig. 7.

Figure 7a–c ) depict the sensor deployment schemes for coverage requirements of 80%, 90%, and 99%, respectively, with a sensing radius $R = 125$. These results align well with our expectations, and the deployment scheme in Fig. 7c is particularly similar to that obtained using the CGD initialization method, indicating a highly efficient coverage strategy. Figure 7d–f show the sensor deployment schemes for coverage requirements of 80%, 90%, and 99%, respectively, with a sensing radius $R = 100$.

Notably, the results in Fig. 7f deserve special attention. When compared to Scenario 2 of Experiment 1, both experiments have the same number of nodes (40), but the final coverage rate in Scenario 2 of Experiment 1 is 98.54%, while the result in Fig. 7f achieves 99.03%. We conducted multiple simulation tests to verify that this difference is not a random occurrence. The key distinction between the two experiments lies in the use of a dynamic node adjustment strategy in Experiment 2. This dynamic adjustment allows the EHPSO algorithm to escape local optima more effectively, whereas the EHPSO algorithm in Experiment 1, lacking this dynamic adjustment, struggles to do so.

**Fig. 8:**
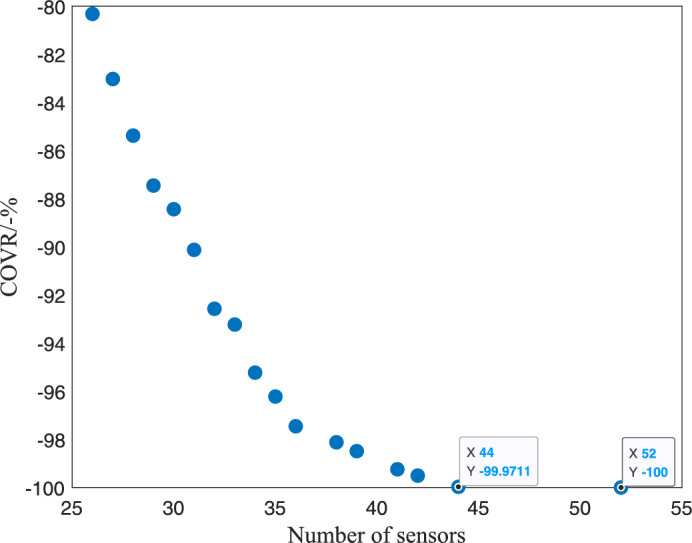
Pareto front obtained by the MOPSO algorithm.

To further illustrate the EHPSO algorithm’s performance and its effectiveness in balancing the number of sensors and coverage rate, we evaluated the MOPSO algorithm (using OBJ-III as the objective function) under a sensing radius of 100 meters. The Pareto front obtained from the MOPSO algorithm is depicted in Fig. 8, where each point on the front represents an optimal trade-off between the number of deployed sensors and the achieved coverage rate. There is a clear positive correlation between the number of sensors deployed and the coverage rate achieved.

Specifically, as the number of sensors increases from 26 to 36, the coverage rate rises almost proportionally from 80.32 to 97.45%. When the number of sensors further increases from 36 to 44, the coverage rate also rises almost proportionally from 97.45 to 99.97%, but at a slower rate compared to the increase from 26 to 36 sensors.

In Fig. 8, we have highlighted two specific points: (44, $-99.9711$) and (52, $-100$). These points indicate that to achieve full coverage (100%), an additional 8 sensors are required beyond the 44 sensors needed for a 99.97% coverage rate. This suggests that ensuring complete coverage without any coverage holes remains an area that requires further investigation.

The results of Experiment 2 highlight the effectiveness of the EHPSO algorithm in dynamically adjusting the number of sensor nodes to meet different coverage requirements.

### Experiment 4: impact of RA and NCA on coverage performance

**Fig. 9:**
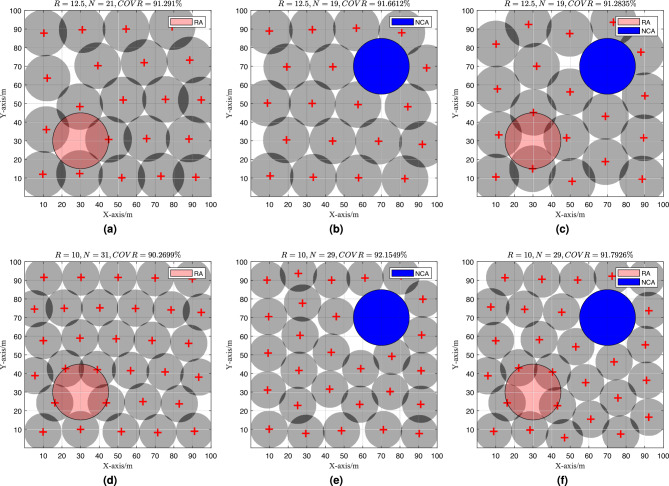
Coverage optimization effect of EHPSO for different RA and NCA conditions.

Three experiments were conducted to investigate the impact of NCA and RA on the EHPSO algorithm. The first experiment included one NCA region, the second experiment contained one RA region, and the third experiment incorporated both one NCA and one RA region. In these experiments, both the NCA and RA were circular regions with a radius of 15 meters. The center of the RA region was located at coordinates (30, 30), and the center of the NCA region was located at coordinates (70, 70). The target coverage rate $C_{\text {obj}}$ was set to 90%. The experimental results are shown in Fig. 9, where the RA region is represented by a red circle and the NCA region is represented by a blue circle.

From the experimental results, it is evident that the EHPSO algorithm can effectively avoid deploying sensors in the RA region and minimize the overlap between the sensing regions and the NCA region. This approach improves the utilization of sensors and reduces energy consumption.

To further investigate the impact of RA and NCA regions on WSN coverage, we compared the optimization results obtained by the EHPSO algorithm in six scenarios from Experiment 2 (Fig. 5) and six scenarios from Experiment 3. The results are summarized in Table 4.

**Table 4:** Summary of optimization results.

No.	R (m)	N	COVR (%)	With RA	With NCA
1	12.5	21	92.61	No	No
2	12.5	21	91.29	Yes	No
3	12.5	19	91.66	No	Yes
4	12.5	19	91.28	Yes	Yes
5	10	31	90.75	No	No
6	10	31	90.27	Yes	No
7	10	29	92.15	No	Yes
8	10	29	91.79	Yes	Yes

The results of Experiment 3 provide valuable insights into the impact of NCA and RA regions on the EHPSO algorithm’s performance:

- Comparison of Experiments No. 1 and No. 2: The presence of an RA region affects sensor deployment. When the number of sensors is the same, the network’s sensing coverage rate decreases. For example, in Experiment No. 1 (without RA), the coverage rate is 92.61%, while in Experiment No. 2 (with RA), it is 91.29%.
- Comparison of Experiments No. 1 and No. 3: The presence of an NCA region reduces the coverage requirement in the task area. By appropriately setting NCA regions, the number of sensors required to achieve the same coverage rate can be reduced. For instance, in Experiment No. 1 (without NCA), 21 sensors are needed to achieve 92.61% coverage, while in Experiment No. 3 (with NCA), only 19 sensors are needed to achieve 91.66% coverage.
- Comparison of Experiments No. 1 and No. 4: The combined presence of both RA and NCA regions further influences the deployment strategy. In Experiment No. 4, the coverage rate is 91.28% with 19 sensors, slightly lower than Experiment No. 1 but with fewer sensors.

The same trends are observed in the experiments with a sensing radius of 10 m (Experiments No. 5 to No. 8). These results confirm that the EHPSO algorithm can effectively adapt to the presence of RA and NCA regions, optimizing sensor deployment to meet coverage requirements while minimizing energy consumption.

## Conclusion and future work

To address the coverage optimization problem in WSNs, we proposed the EHPSO algorithm, aiming to achieve the requirement coverage threshold while minimizing the number of sensors. We conducted three experiments to evaluate the algorithm’s performance. The first two experiments demonstrated that the EHPSO algorithm performs well in different task area sizes, achieving high coverage rates with fewer sensors. The third experiment investigated the impact of RA and NCA on WSN coverage. The results showed that RA regions increase the difficulty of achieving the desired coverage, while appropriate NCA regions can reduce this difficulty. These findings highlight the flexibility and effectiveness of the EHPSO algorithm in optimizing WSN coverage.

Although the EHPSO algorithm can provide cost-effective deployment strategies for WSNs, there are still some research issues and challenges to be addressed in the future:

- The transmission protocols among sensing nodes are not discussed in this paper. For example, how to establish energy-efficient routing between nodes could be further explored by employing techniques such as compressive sensing and designing appropriate routing strategies among sensor nodes to reduce overall energy consumption^40^. Additionally, clustering methods among sensing nodes are also worth investigating. Due to issues such as channel fading, insufficient, and random energy arrivals in WSNs, it is important to group sensor nodes into appropriate sub-networks to improve network throughput^41^. Moreover, other critical aspects such as data aggregation mechanisms across different sensors and duty cycle scheduling^42^ also require further study. These topics need to be addressed in future work in order to achieve more efficient energy management in WSNs.
- As analyzed in the “Complexity Analysis” section, the main source of computational complexity lies in the fitness evaluation function. When more complex RA and NCA regions are considered or the number of deployed sensors increases significantly, the complexity of the coverage evaluation grows rapidly. Therefore, designing a more efficient coverage evaluation function is one of our planned future research directions. We believe that an adaptive grid-based approach could be a promising solution^43,44^, where the grid size is dynamically adjusted during the search process. Specifically, a coarse initial solution can be obtained quickly with larger grids at the early stage of the algorithm, followed by finer searches using smaller grids for refinement. In addition, although EHPSO achieves better performance compared to traditional algorithms, it comes with slightly increased computational complexity. Since EHPSO is built upon the PSO framework and many parallel implementations of PSO have been studied, we plan to explore parallel computing techniques in future work to reduce the runtime of the algorithm.
- Finally, regarding the issue of 100% coverage, there are indeed real-world scenarios where WSNs are required to achieve full (or even over 100%) coverage. In such cases, the focus shifts toward avoiding coverage holes, which differs somewhat from the problem setting in this paper. This type of scenario is more closely related to the k-coverage problem. We believe that the proposed EHPSO algorithm has strong potential for application in such k-coverage problems and will explore its effectiveness in these settings in future research.

## Data Availability

The datasets generated and analysed during the current study are available in the github repository (https://github.com/linjfcl/EHPSO.git).
